# A Lipidomic Approach to Understanding Free Fatty Acid Lipogenesis Derived from Dissolved Inorganic Carbon within Cnidarian-Dinoflagellate Symbiosis

**DOI:** 10.1371/journal.pone.0046801

**Published:** 2012-10-24

**Authors:** Simon R. Dunn, Michael C. Thomas, Geoffrey W. Nette, Sophie G. Dove

**Affiliations:** 1 ARC Centre of Excellence for Coral Reef Studies, Global Change Institute, University of Queensland, St. Lucia, Brisbane, Queensland, Australia; 2 Independent Marine Biochemical Research, Moreton Bay Research Station, Dunwich, Queensland, Australia; King Abdullah University of Science and Technology, Saudi Arabia

## Abstract

The cnidarian-dinoflagellate symbiosis is arguably one of the most important within the marine environment in that it is integral to the formation of coral reefs. However, the regulatory processes that perpetuate this symbiosis remain unresolved. It is essential to understand these processes, if we are to elucidate the mechanisms that support growth and resource accumulation by coral host, and conversely, recently observed reduction and/or mortality of corals in response to rapid environmental change. This study specifically focused on one area of metabolic activity within the symbiosis, that of free fatty acid synthesis within both the dinoflagellate symbionts and cnidarian host. The main model system used was *Aiptasia pulchella* and *Symbiodinium* sp. in combination with aposymbiotic *A. pulchella*, the symbiotic coral *Acropora millepora* system and dinoflagellate culture. Fatty acids (FAs) were selected because of their multiple essential roles inclusive of energy storage (resource accumulation), membrane structure fluidity and cell signaling. The study addressed free FA lipogenesis by using a new method of enriched stable isotopic (^13^C) incorporation from dissolved inorganic carbon (DI^13^C) combined with HPLC-MS. FAs derived from DI^13^C aligned with a mixture of known lipogenesis pathways with the addition of some unusual FAs. After 120 hr, ^13^C-enriched FA synthesis rates were attributed to only a complex integration of both *n*–3 and *n*–6 lipogenesis pathways within the dinoflagellate symbionts. Furthermore, there was no detectible evidence of symbiont derived enriched isotope fatty acids, catabolized ^13^C derivatives or DI^13^C being directly utilized, in host late *n*–6 pathway long-chain FA lipogenesis. These findings do not align with a popular mutualistic translocation model with respect to the use of translocated symbiont photoassimilates in host long-chain FA lipogenesis, which has important connotations for linking nutrient sources with metabolite production and the dynamic regulation of this symbiosis.

## Introduction

Lipids, in particular fatty acids (FAs), are essential to cell and metabolic function and are typically associated with energy storage and structural fluidity of membranes. In addition, FAs form multiple complex compounds, which have important roles in cell signaling processes [1], [2]. FAs have a carbon based ‘backbone’ chain to their molecular structure, which may be elongated, shortened or altered by either elongase or desaturase enzymic activity respectively. As FA synthesis proceeds along a lipogenesis pathway, the longer a FA carbon chain will be. The size of the FA structure is also dependent upon the addition or removal of double hydrogen bonds and catabolic activity. The complement of lipogenesis pathways and associated enzymes, vary between animals (*n* (or ω)-6 pathway), plants (*n* (or ω)-3 pathway) and algae (both *n*–3 and *n*–6 pathways) resulting in a differential capacity to produce FAs within different organisms. The absence of certain functional lipogenesis pathways in animals can result in FA deficiency and affect health. However, an inability to synthesize FAs that are essential to animals, such as linoleic acid and docosahexaenoic acid (DHA), can be resolved by obtaining them from dietary sources [3]. For example, late lipogenesis pathway *n*(ω)-3 FAs including ‘essential’ FAs, such as DHA, are synthesized by marine algae, but animals with the *n*(ω)-6 lipogenesis pathway generally do not have the complement of enzymes to produce endogenous DHA [2]. Typically, animals acquire DHA through a food chain linked to marine algae or plants with the *n*–3 pathway. An alternative form of sequestering metabolites including lipids is through a symbiotic interaction with organisms that are able to synthesize the essential metabolites. In this case, metabolites including essential products are translocated and utilized by one or both partner organisms [4]. Specifically, FA translocation has been implied or reported to occur from the symbiotic dinoflagellates into a variety of hosts such as, the giant clams, tridacnids [5], and cnidarians including anthozoans [6] and scyphozoans [7]. However, the rates of specific metabolite synthesis resulting from translocation were not provided in these studies.

The high metabolic productivity of dinoflagellates residing within the coral (Anthozoa, Cnidaria) host is believed to contribute to the formation of coral reefs through the translocation of carbon rich products from the symbiont to the host. Lipids are the main energy store in cnidarians and the primary products derived from photosynthetically-fixed carbon translocated from the dinoflagellate symbionts to the host [8]. Total lipids from the symbiosis (triacylglycerols, wax esters, phospholipids and free FAs), account for 10–46% of the cnidarian tissue dry weight [9], [10]. Previous studies reported that metabolites, including lipids vary in composition and abundance according to the carbon source and species [11], [12]. Furthermore, changes in environmental stimuli such as light and temperature, elicit a marked change in lipid production and subsequently the potential storage and availability of energy for coral-dinoflagellate symbiosis, which has indirect implications for reef health [9], [13], [14]. Photoautotrophs rely upon CO_2_ diffusion for organic carbon fixation using the enzyme ribulose-1,5-bisphosphate carboxylase/oxygenase (RUBISCO) to produce metabolites such as sugars, amino acids, proteins and lipids. In the marine environment, photoautotrophs have a limited availability of dissolved CO_2_ for photosynthesis due to ambient seawater pH and associated gaseous saturation states [15], [16]. The dominant form of dissolved inorganic carbon (DIC) in the marine environment is bicarbonate (HCO_3_^−^; ∼95% of the total DIC in seawater at ∼pH 8.2) and in order for marine photoautotrophs, such as Chlorophyta and Dinophyta, to access and utilize the more abundant form of DIC, photoautotrophs use carbon-concentrating mechanisms (CCMs) such as the enzyme, carbonic anhydrase (CA). CA catalyzes the reversible conversion between HCO_3_^-^ and CO_2_, to allow diffusion of DIC across bipolar membranes and supplement limited ambient CO_2_ for photosynthesis and enable RUBISCO to function efficiently [16]. A combination of respiration and CA activity in cnidarian hosts of symbiotic dinoflagellates [17] are important CO_2_ supplies for symbiont metabolite production [18], which in part, meet the energy requirements of both the symbiont and host [15], [19].

Understanding the complex metabolic contribution of each partner within a symbiotic interaction requires the translocation and rates of specific metabolite production to be determined. Previous ^14^C radioisotope studies have indicated direction of transfer into either, or both the host and symbiont, as well as the rate of carbon fixation from DIC source to total products [11], [20]. In other studies, ^14^C incubations from 5–60 min to several hours have shown that photosynthetic fixation occurs rapidly, resulting in, almost immediate incorporation into both host and symbiont products, including total lipids [20], [21], [22], [23]. However, the use of ^14^C is problematic because of radioisotope regulations, safe work practise and difficulties associated with using radioisotopes within protected environments. In addition, although detection of radioisotope incorporation into metabolites may suggest a carbon source to a product, there may be potential resolution restrictions for identification and measurement of metabolite synthesis rates when compared to the application of alternative techniques. In previous studies the use of ^14^C may also underestimate translocation by up to 50% [24]. For example, short-term isotopic incubation may clearly show rapid synthesis and translocation of one form of metabolite over another that may take longer to accumulate. The resulting and subsequent extrapolation may infer importance of that metabolite over another form, which may actually not be the case. For example, Whitehead and Douglas [23], clearly showed with short-term ^14^C incubations, rapid synthesis and translocation of trichloroacetic acid soluble compounds such as the sugar, glucose and citric acid cycle compound, succinate between symbiont and host. These translocated metabolites were in relatively large quantities compared to “barely detectable” levels of lipid incorporation, which were dependent on algal photosynthesis. Whitehead and Douglas [23] as a result, proposed the results of the study did not represent complete translocation, which may be subject to temporal variability in synthesis rates and subsequent potential delayed translocation of other metabolites, such as lipids.

Tracking a source of carbon to specific metabolite synthesis may benefit from the application of an alternative technique. Sequestration of carbon from different sources can be characterized by the isotopic profile of mixed ^12^C and ^13^C proportions. For example, the isotopic ratio of carbon fixation via a phototrophic dinoflagellate is subtly different to that of the animal host via heterotrophy and by measuring the ratio of ^13^C:^12^C can indicate the trophic source of a compound through a carbon ratio signature [25], [26], [27], [28], [29]. In naturally occurring organic compounds, the isotopic fraction ratio of ^13^C to ^12^C is approximately 1–2% irrespective of source [13], [30], and naturally-occurring multiple isotopologues containing ^13^C (>1–2%) are of low abundance. By increasing the isotopic fraction available for fixation, through an enrichment of a source such as DI^13^C, the likelihood of ^13^C incorporation thus increases beyond the naturally-occurring 1–2% and subsequently can act as a biomarker for metabolite synthesis.

Here, we introduce an alternative isotopic method to the use of radioisotopes to track a carbon source into sequential incorporation of metabolites. Specifically, this technique is favoured over using GC-MS (and its inherent greater level of molecular fractionation) for the following three reasons. Firstly, as there is no inherent fragmentation for each measured FA, an array of isotopologues that illustrate a distribution of different isotopic carbon within molecular structures, that can also be used to measure rates of synthesis. Secondly, if so required, a specific target ion can be selected for molecular structural determination and verification. Thirdly, samples do not require derivatization and can be directly analysed from the extraction. This study focused upon the lipogenesis of free FAs from a known source and concentration of DIC to determine which pathways are active within each organism that is using the DIC source in FA synthesis, the rate of synthesis, and the potential of translocation between organisms and subsequent further use in this symbiosis. The symbiotic anemone, *Aiptasia pulchella* (Anthozoa, Cnidaria) was chosen as a model symbiotic cnidarian-dinoflagellate consortium for this study, for two main reasons: Firstly, large cultures can be maintained easily and individuals of a uniform size can be specifically selected for experiments to minimize potentially variability in metabolism relating to different size. Secondly, *A. pulchella* can be maintained in a healthy aposymbiotic state and therefore offers an ideal control for experiments focused upon the symbiosis with the dinoflagellate. However, to assess the method transferability, a separate scleractinian hermatypic coral model and an algal culture were also used to demonstrate the broader application of the technique described here.

## Materials and Methods

### Culture Conditions

*Aiptasia pulchella* were initially supplied through a local aquarium supplier. The cultures of the symbiotic sea anemone, A pulchella were maintained at Moreton Bay Research Station (MBRS), North Stradbroke Island, QLD under a 12 hr light (350–400 µmol m^−2^ s^−1^ from a metal halide unit)/12 hr dark regime for over a year. The culture temperature was maintained at 25–26°C. Anemones were fed once a week with frozen brine shrimp nauplii. Aposymbiotic anemones were produced using an adapted cold stripping method to remove symbiotic dinoflagellates [31] and then maintained in the dark for over 4 months with two monthly repeated stripping applications during that period.

### ^13^C Isotope Incubation

The stable isotope ^13^C in the form of NaH^13^CO_3_ (Sigma-Aldrich) was used in an artificial seawater (ASW) mixture adapted from Harrison et al [32]. The final concentration of dissolved inorganic ^13^ carbon (DI^13^C) at 2 mM corresponded to that of normal seawater at pH 8.2, 33–34 ppt. Control ASW used non-isotopic NaH^12^CO_3_ in the same quantity. Anemones were removed from culture conditions and placed in separate polycarbonate chambers containing ^13^C-enriched ASW, under normal culture light and temperature conditions (n = 5 symbiotic and n = 3 aposymbiotic for each extraction point). Different individual symbiotic anemones were placed in control environments for the same experimental duration (n = 5 for each extraction point). The different ASW treatments were replaced every 24 hr. The anemones were fed once four days prior to, but not, during experimental treatments. Sample anemones and any expelled dinoflagellate pellets were removed at 24 hr, 48 hr, 72 hr and 120 hr from ASW treatments, snap frozen in liquid nitrogen and then stored at −80°C prior to processing. The respective expelled dinoflagellate pellets from the ^13^C-enriched ASW treatment were collected at each time point, but due to low quantities were later combined to provide an integrated measurement over the total experiment duration from 0 to 120 hr prior to processing.

### Lipid and Fatty Acid Extraction

Sampled anemones and dinoflagellates previously stored at −80°C were thawed and placed into an RVC2-18 (Christ) speed vac connected to a MZ2C (Vacuubrand) vacuum pump and desiccated to a dried pellet overnight. The dried pellets of each sample were then weighed. The total lipid fraction from each pellet was extracted using modified methods of Folch et al [33]. Each individual pellet was placed into a glass homogenizer together with 2 mL of fresh chloroform/methanol (2∶1) and homogenized. The homogenate for each individual sample was then returned to a 15 mL falcon tube. The homogenizer was rinsed with 2 mL of chloroform/methanol (2∶1) and added to the original homogenate in a 15 mL falcon tube, vortexed and stored for 2 hr in the dark at 4°C. The chilled suspension was removed and filtered using a 22 µm glass fibre filter and placed in a new 15 mL falcon tube. The filter was then rinsed with 1 mL of chloroform/methanol (2∶1), which was added to the filtered homogenised sample. 1 mL of 0.1 M KCl in Milli-Q water was added to the combined 5 mL of homogenate filtered extract, vortexed and placed in the dark at 4°C for 1 hr or until the aqueous and organic phases had separated. The aqueous phase was removed and remaining organic phase washed with 5 mL of methanol/water (1∶1), the phases were allowed to separate for 1 hr before the aqueous phase was removed and the process repeated (x3). Following the final rinse the organic phase was dried, weighed and the resulting pellet resuspended in 500 µL of 100% acetonitrile (B and J Brand HPLC grade, Lomb Scientific) with the aid of a 1.5 mL tube tissue homogenizer (Eppendorf).

### High Performance Liquid Chromatography – Mass Spectrometry (HPLC-MS) of Fatty Acids

The FAs from the total lipid extracts were analyzed using an Agilent 1200 Series HPLC instrument (Agilent Technologies, Inc., Santa Clara, USA) coupled to an LTQ XL linear ion trap mass spectrometer (Thermo Fisher Scientific, Waltham, USA). In addition to the HPLC unit, the following components were installed: micro degasser (G1379B), binary pump SL (G1312B), high performance autosampler SL (G1367C) thermostat column compartment SL (G1316B) and a diode array detector SL (G1315C). Chromatography was performed using a Luna 3µ C18 (2) 100 Å 75×4.60 mm column (Phenomenex, Torrance, USA). Milli-Q water and 100% acetonitrile were used as the A1 and B1 solvents, respectively. For sample analysis, 10 µL of each lipid extract sample was injected using the autosampler. The components of each extraction were analyzed using a 40 min run with a HPLC flow rate of 0.8 mL/min. The gradient elution profile used during each analysis run is shown in Table S1.

The LTQ XL mass spectrometer was equipped with an Ion Max electrospray ion source and run in negative ion mode with the following instrument settings: spray voltage −4.5 kV, capillary voltage −130 V, tube voltage 100 V and capillary temperature 320°C. The nitrogen flow rates at the ESI source were set as follows: sheath gas 32 (arbitrary units) and auxiliary gas 8 (arbitrary units). No sweep gas was used. Nitrogen was sourced using a Domnick Hunter LCMS30-1-E nitrogen generator (Parker Hannifin Ltd, Industrial Division, England). The Luna C18 column was subjected to a cleaning regime every 10 sample runs with a 1∶1 mixture of ethanol and hexane for approximately 20 min to remove non-specific highly-hydrophobic compounds within sample extracts, which do not elute from the column using 100% acetonitrile.

A series of FA standards (Sapphire Bioscience, Waterloo, NSW, Australia) were used to identify retention times (*t*_R_) of specific *m/z* profiles associated with known FAs (Table S2). In previous trials, MS spectra were analyzed within a mass range of *m/z* 200–1000 (data not shown), however no FA signals and ion profiles of interest to this study were observed above *m/z* 500. Consequently, for data collection the MS spectra were recorded during the entire 40 min HPLC runs within a mass range of *m/z* 200–550. Spectra were recorded in profile mode with a maximum ion injection time of 50 ms and an automatic gain control setting of 3.00e+04. The number of microscans was set at 4 to minimize the file size of the MS data. The percentage of each isotopologue by number of ions was determined from the MS spectrum by converting the peak areas of each isotopologue into a relative percentage (normalized abundance). The peak span measurement was taken at 50% across FA peak heights on the ion chromatogram. The isotopologues containing the additional ^13^C stable isotope was then added to give a measurement of total ^13^C incorporation for a given time point. Isotopologues were verified as being associated with the respective FA by displaying the same elution profile and by the use of collision-induced dissociation for selected FA ion profiles (data not shown). In addition, the observed distributions of ^13^C-labeled isotopologues were clearly matched to the theoretical distributions predicted mathematically.

### Quantification of Extraction Efficiency in Negative Ion MS/MS

In order to determine whether the described method resulted in the same extraction efficiency of FAs from the total lipids from different samples of anemones of different symbiotic states, the quantitation of arachidonic acid (AA) was used as a traceable marker. The sample analysis run time was reduced from 40 min to 15 min and the gradient elution was adapted accordingly (Table S3) using a flow rate of 0.8 mL/min. The MS/MS of the AA [M-H]^−^ ion at *m/z* 303.6 was performed with a normalized collision energy of 22% and an isolation width of 1 Th. The max injection time was changed to 30 ms and the automatic gain control was set to 10000. MS/MS spectra were acquired from *m/z* 80–320. The HPLC-MS instrument settings were reset to a source voltage of 3.2 kV, sheath gas flow rate 40 (arbitrary units), auxiliary gas flow rate 20 (arbitrary units), capillary voltage −28 V, capillary temperature 320°C and tube lens voltage −75 V. The ion chromatogram for the *m/z* 259.3 fragment ion (carbon dioxide neutral loss) was displayed using a range of *m/z* 258.8–259.8. The area of the chromatographic peak corresponding to AA (*t*_R_  = 5.6 min) was integrated using the genesis algorithm without the use of a prior smoothing function within the Xcalibur ™software (Vers. 2.0.7, Thermo Scientific).

For quantification, a standard curve of AA concentration was produced for comparison with the quantity of AA in experimental samples. A dilution gradient of AA standard (Sapphire Bioscience, Waterloo, NSW, Australia) was produced in that 5 µL injection volumes resulted in quantities of 156.25, 312.5, 625 and 1250 pg, respectively. A series of *A. pulchella* samples from the 72 hr incubation were diluted 1∶200 and repeatedly run alternately to standard dilutions such that any drift in analysis response could be both observed and corrected for. Two standard curves were constructed by averaging the peak areas for run 1 & 2 and 2 & 3 for the AA dilution series. The amount of AA within the samples and standards was verified from a linear trendline formulae and the amount of AA in 5 µL (the injection volume) of the 1∶200 diluted *A. pulchella* samples. The amount of AA was then converted to a concentration and adjusted to determine the AA quantity in undiluted samples.

Octadeuterated arachidonic acid (D8-AA) (Sapphire Bioscience, Waterloo, NSW, Australia) was used as a traceable marker throughout the extraction process to measure the extraction efficiency of the FAs from the total lipids. The D_8_-AA standard was added to tissue homogenate at a final concentration of 0.8 ng/µL prior to extraction and then compared to the known concentration of AA (see above) within each sample following the extraction. The D_8_-AA, which has eight deuterium atoms within its molecular structure, has a distinct isotopologue mass profile compared to the endogenous AA. D_8_-AA has an *m/z* of 311, however, ions of *m/z* 306–310 ions were also observed in the ESI-MS spectrum of the deuterium-labeled standard. The *m/z* 310 ion was the most abundant and likely represents the D7-AA isotopologue. It is assumed that the ionization efficiency of AA and the D_8_-AA standard are the same, since the isotopologues are chemically identical (although a very small difference in retention times is observed between different deuterated isotopologues). For a direct comparison of endogenous AA to the D_8_-AA standard, the *m/z* 303 ion of AA and the *m/z* 310 ion of the D_8_-AA standard were converted to mole fractions. Within an AA standard, the *m/*z 303 ion was calculated to have a mole fraction of 0.80. For the D_8_-AA standard, the mole fraction of the *m/z* 310 ion was determined to be 0.31. The chromatograms for the *m/*z 303 and 310 from a LC-MS spectrum of a sample containing both endogenous AA and D_8_-AA standard were integrated with the genesis function without prior smoothing. The peak area for the *m/*z 303 ion was then divided by the mole fraction 0.80 to give a peak area representative of all the isotopologues of AA (AA peak area). This process was repeated for the *m/z* 310 ion of the D8-AA standard using the mole fraction of 0.31 to give a peak area representative of all the isotopologues of D_8_-AA (D_8_-AA peak area). The previously known concentration of AA within an experimental sample was converted from ng/µL to molarity and used to calculate the molarity of the D_8_-AA by multiplying the molarity of AA by D_8_-AA peak area/AA peak area. The molarity calculated for D_8_-AA was then converted to ng/µL using the molecular mass of D_8_-AA. The measurement of extraction efficiency was then calculated by dividing the measured D_8_-AA concentration by the pre-extraction known concentration (0.8 ng/µL) and expressed as a percentage.

The FAs with increased ^13^C profiles were compared to known lipogenesis pathways from the online databases (Lipid library (www.lipidlibrary.aocs.org), lipidmaps (http://www.lipidmaps.org) and KEGG 1.3 lipid pathways (www.genome.jp/kegg/pathway).

### Transferability of Methods: Coral and Dinoflagellate Culture Model Systems

To assess transferability of the DI^13^C incorporation method for other model systems that utilize dissolved HCO_3_^-^ as a carbon source for metabolite synthesis and specifically lipogenesis the hermatypic coral *Acropora millepora* (Anthozoa, Cnidaria) complete with integral clade C3 symbionts and a separate clade B1 dinoflagellate culture were incubated in the same ^13^C enriched ASW as the *A. pulchella* model for 48 hr. Clade B1 culture was chosen as a comparable model culture as this clade is known to form a symbiosis with *A. pulchella* [34]. The incubation method for *A. pulchella* was adapted to accommodate the differences in the model systems as described below.

#### The hermatypic coral, acropora millepora model

For the *A. millepora* model, an area of approximately 3×10 cm Ø at the tips of the corymbose branches (8–10 main branches) from three colonies approximately 50 cm across was removed and immediately placed in polycarbonate chambers containing the DI^13^C enriched ASW, and partly immersed in flow-through outdoor aquaria. Portions of coral colonies were removed from Heron Island Reef flat, Great Barrier Reef, Australia (see acknowledgements for permit details). The enriched ^13^C ASW was exchanged every 12 hr for a period of 48 hr and maintained in ambient light and temperature conditions (1100–2000 µmol m^−2^ s^−1^; 26–27°C). The cluster of branches was then removed and frozen in liquid N_2_ for later processing. The processing method was adapted from the *A. pulchella* model to account for removal of tissue from the skeleton whereby a frozen 2 cm length of one branch of each individual colony was incubated in the chloroform/methanol extraction mixture in the dark overnight at 4°C. The remainder of the extraction protocol was as previously described for *A. pulchella*.

#### Dinoflagellate clade B1 culture

Clade B1 dinoflagellate cultures were maintained in accordance with Rosic and Hoegh-Guldberg [35]. The method of ^13^C incorporation was adapted for use with the culture model by mixing the F2 media (Guillard’s Marine Water Enrichment – Solution, Sigma) with the ^13^C enriched ASW as used with the *A. pulchella* model. After 48 hr the enriched treated culture were removed from the media by centrifugation at 2775 RCF for 5 min. Following removal of the supernatant, the pellet was resuspended in ASW and re-centrifuged to rinse excess enriched media. Following an additional rinse the culture was centrifuged at 9200×g for 10 min and the pellet frozen in liquid N_2_ for later processing. The culture pellets were then treated in the same way as for the *A. pulchella* extractions.

#### Statistics

All statistical test comparisons were undertaken with Minitab™ v16 software. Two-tail paired *t*-tests were performed on total arachidonic acid concentration and total lipid extraction efficiencies. Following Anderson-Darling tests for normality and box-plot tests for heteroscedasticity, one-way ANOVA tests were performed with multiple tukey pairwise post-hoc comparisons for the isotopic incorporation (n = 4) over time. Tukey post-hoc comparisons were tested to set significance values of P<0.05 and P<0.01 as part of the program. A two-tail two-sample *t*-test was used to test between treatments where an observed difference between controls was noted.

## Results

### Extraction Efficiency and Arachidonic Acid Concentration

The outcomes of the comparative deuterated (D8) and non-deuterated AA concentration extraction efficiency method from total lipids, showed no difference between aposymbiotic and symbiotic anemones (Figure S1A and B).

### Stable Isotope Incorporation during Lipogenesis

#### Controls

**Figure 1:**
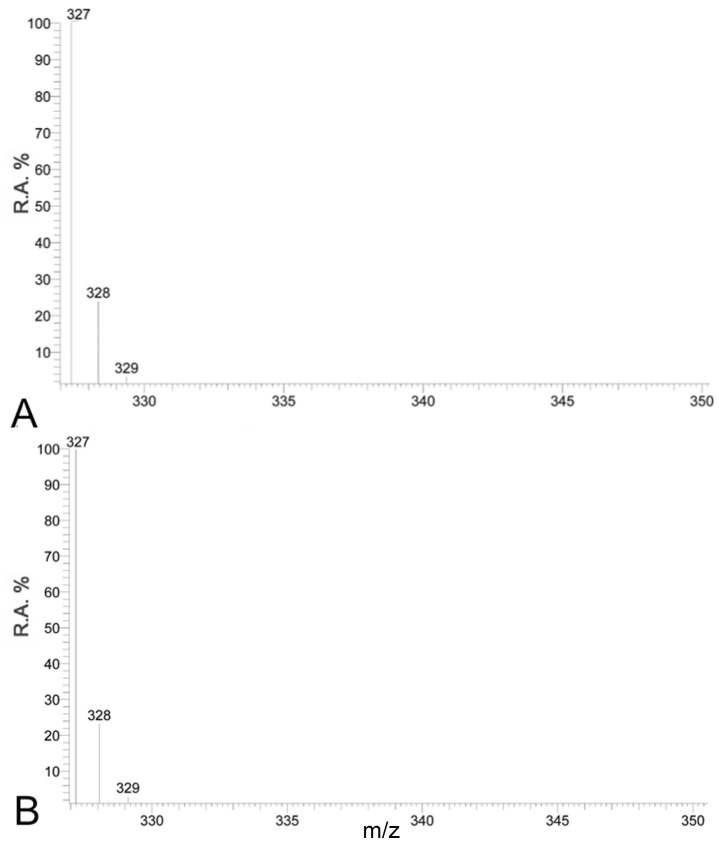
The spectrum profile of the fatty acid, DHA (C_22∶6, __*n*–3_) within (A) a aposymbiotic *Aiptasia pulchella* removed from the DI^13^C ASW at 72 hr and (B) within a symbiotic *A. pulchella* removed from the non-^13^C DIC ASW at 72 hr. (R.A.%  =  Relative Amount).

**Figure 2:**
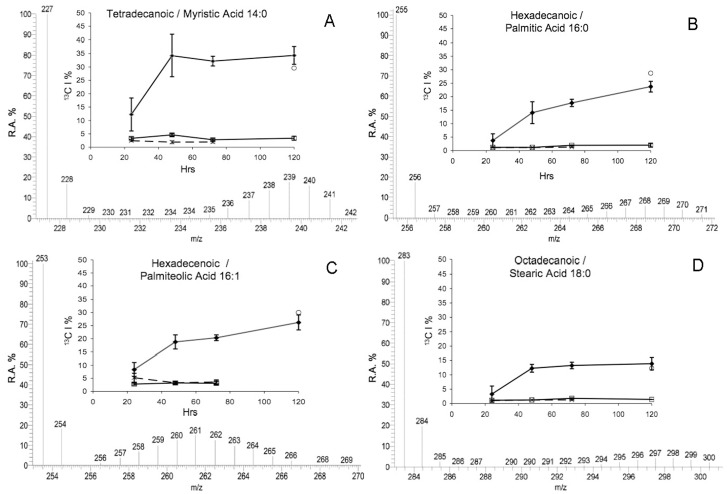
Isotopic incorporation lipid profiles of fatty acids in symbiotic *A. pulchella*. Subplots: Relative Amount (R.A.) of ^13^C incorporation (^13^C I) into the FA structures (%) over time. Key: R.A.%  =  Relative Abundance of isotopologues, m/z  =  Mass-to-Charge ratio of isotopologues, ^13^CI %  =  Isotopic incorporation in isotopologues, ⧫  =  DI^13^C ASW treated symbiotic anemones (n = 5), Square symbol  =  DI^13^C ASW treated aposymbiotic anemones (n = 3), X  =  Non-isotopic DIC ASW treated symbiotic anemones (n = 5), Circle symbol  =  DI^13^C ASW Dinoflagellates expelled from treated symbiotic anemones (combined from treatment over time). Error Bars  =  Standard Deviation. Note: Where data not shown for time points  =  Signal was below detection. Spectrum data shown is from DI^13^C ASW symbiotic anemones at 72 hr.

**Figure 3:**
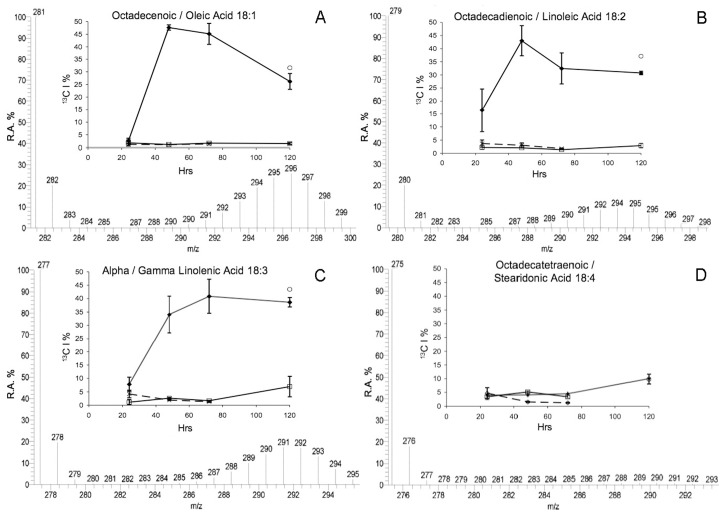
See **Figure 2** legend.

**Figure 4:**
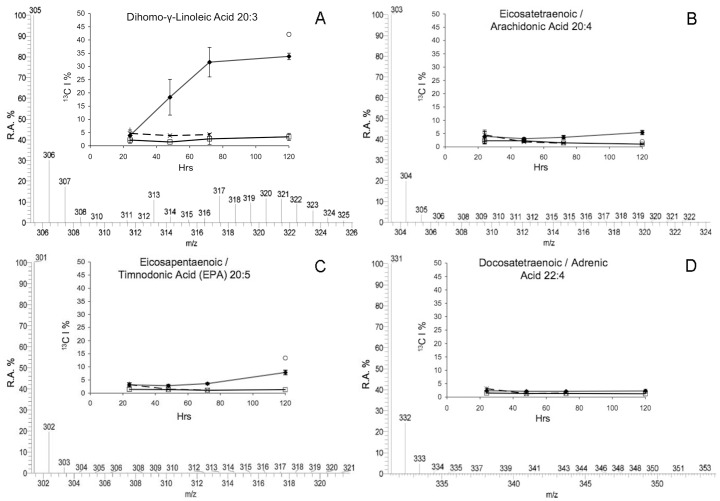
See **Figure 2** legend.

**Figure 5:**
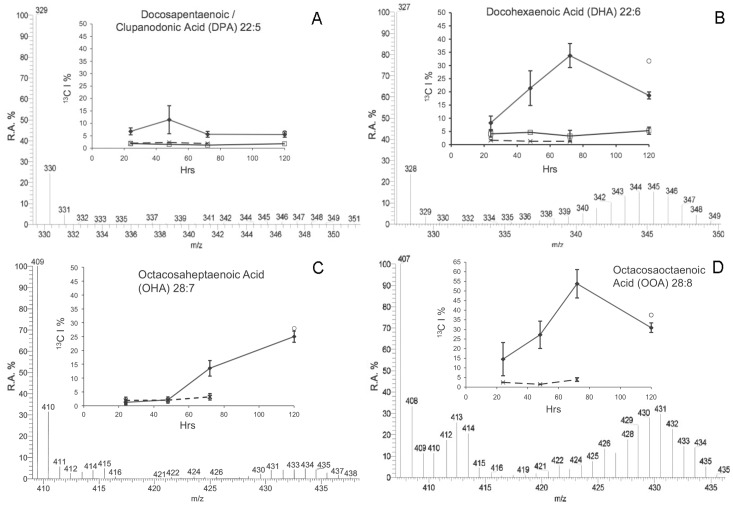
See **Figure 2** legend.

A naturally occurring ^13^C incorporation of approximately 1–2% was detected within all FAs from different samples. There was no additional ^13^C incorporation in FAs from either DI^13^C ASW treated aposymbiotic anemones, or non-enriched DIC ASW treated symbiotic anemones as highlighted by the absence of additional isotopologues of docosahexaenoic acid (DHA C_22∶6_: *m/z* 327) (Figure 1A and B) or FA lipogenesis profiles over time (Figure 2, 3, 4, and 5).

#### Enriched DI^13^C media treated symbiotic A. pulchella and expelled dinoflagellates

Apart from very long chain fatty acids (LCFA), the same FAs were detected in all samples. However, incorporation of enriched ^13^C was only detected into different FAs within both symbiotic anemones and expelled dinoflagellates collected from the enriched DI^13^C ASW, which also varied between FAs over time. During FA synthesis the ^13^C was incorporated in different ways according to the FA, and was either an immediate and rapid incorporation with a V_max_ between 48 hr and 72 hr followed by a plateau profile, or a continuous linear incorporation over time, or little/no incorporation at all. Enriched ^13^C isotope incorporation was skewed towards an abundance of isotopologues with a high ^13^C content indicating a dependency upon the DI^13^C rather than an alternate source (^12^C) (Figure 2, 3, 4, and 5) with the exception of two very long-chain FA with a binomial skewed profile (Figure 5C-D).

The FAs derived from enriched ^13^C ranged from tetradecanoic/myristic acid (C_14∶0_ ) to a long-chain FA, octacosaoctaenoic acid (OOA) (C_28∶8_). Specifically, the isotopic incorporation into myristic acid (C_14∶0_ ) within symbiotic anemones increased significantly from 24 hr (*f*  = 18.33, P<0.01) until a V_max_ 34.05% ± Std Dev 8.2 at 48 hr. After 48 hr, the incorporation and synthesis rate was consistent over time (Figure 2A). At 120 hr, the ^13^C incorporation in C_14∶0_ from the cumulative combined expelled dinoflagellate pellets (CEDPs) was 29.58% and similar to symbiotic anemones (34.3% ± Std Dev 3.33)(Figure 2A). The similar isotopic incorporation in FAs measured from the CEDP and symbiotic anemones continued across other FAs. The isotopic incorporation and synthesis rates of palmitic acid (C_16∶0_) and palmiteolic acid (C_16∶1_) were more linear over the total incubation period compared to C_14∶0_ (Figure 2B and 2C). There was a significant increase from 24 hr - 48 hr for C_16∶0_ (*f*  = 59.06, P<0.01) and (C_16∶1_) (*f*  = 49.68, P<0.01). Incorporation steadily increased over time, although the amount at 72 hr was not significantly different to that of 48 hr for both FAs. There was a further significant increase (P<0.05) between 72 hr and 120 hr for both FAs (23.73% ± Std Dev 1.92 for C_16∶0_; Figure 2B and 26.22% ± Std Dev 2.84 for C_16∶1_; Figure 2C). The CEDP incorporation for both C_16∶0_ and C_16∶1_ was marginally greater than symbiotic anemones at 28.75% and 30.02% respectively.

Stearic acid (C_18∶0_) displayed a similar profile to C_14∶0_, albeit with a lower incorporation. V_max_ occurred at 48 hr with a highly significant increase from 24 hr (3.27% ± Std Dev 2.8; *f*  = 31.55, P<0.01) to 48 hr (12.19% ± Std Dev 1.34) followed by no further significant changes in isotopic incorporation (Figure 2D). The same profiles were displayed in the monounsaturated octadecenoic/oleic acid (C_18∶1_), polyunsaturated octadecadienoic/linoleic acid (C_18∶2_) and the α/γ linolenic acid (C_18∶3_) (Figure 3A-C). For the purpose of this study, isotopic profiles of *n–3* and *n–6* derived C_18∶3_ FAs were not analyzed separately. At 48 hr, incorporation significantly increased in C_18∶1_ from 24 hr - 48 hr (2.35% ± Std Dev 0.96 to 47.38% ± Std Dev 4.16; *f*  = 222.63, P<0.01). A reduction in incorporation at 72 hr into C_18∶1_ did not become significant until 120 hr (26.21% ± Std Dev 3.15; P<0.01) and was similar to CEDP at 31.6%. The linoleic acid (C_18∶2_) isotopic incorporation also significantly increased from 24 hr to a V_max_ at 48 hr (43.01% ± Std Dev 5.73; *f*  = 17.63, P<0.01), and like C_18∶1_ was followed by reduced incorporation, which was significant at 120 hr (30.74% ± Std Dev 0.65; P<0.05). At 120 hr, incorporation within C_18∶2_ from CEDP was higher than the symbiotic anemones at 37.12% (Figure 3B). The V_max_ of linolenic acid (C_18∶3_) occurred at 72 hr (40.87% ± Std Dev 6.87). There was a significant increase between 24 hr and 48 hr (*f*  = 47.77, P<0.01) and a decrease between 72 hr and 120 hr. The incorporation in CEDP was, however, similar to the symbiotic anemones at 72 hr (43.44%) (Figure 3C). In contrast to C_18∶3_, the profile for octadecatetraenoic/stearidonic acid (C_18∶4_) displayed very little incorporation in symbiotic anemones over time, until a significant increase from 72 hr (4.53% ± Std Dev 1.27) to 120 hr, (10.05% ± Std Dev 1.33; *f*  = 29.36, P<0.05). The value for the CEDP C_18∶4_ was 9.84% (Figure 3D).

Dihomo-**γ-**linoleic acid (C_20∶3_) synthesis in symbiotic anemones showed a consistent linear rate of incorporation over 72 hr (V_max_ 31.63% ± Std Dev 5.57) with a significant increase between 24 hr to 72 hr (*f*  = 49.1, P<0.01). The incorporation between 72 hr and 120 hr then plateaued, although the CEDP was higher at 42.12% (Figure 4A). The remaining C_20_ fatty acids detected, namely eicosatetraenoic/arachidonic acid (C_20∶4_; AA) and eicosapentaenoic/timnodonic acid (C_20∶5_; EPA) were similar to stearidonic acid (C_18∶4_), with little incorporation over time (Figure 4B and C respectively). There was a slight, but significant increase in symbiotic anemone AA incorporation between 48 hr (3.09% ± Std Dev 0.38) and 120 hr (5.42% ± Std Dev 0.75)(*f*  = 4.21, P<0.05). However, the negligible difference between other time points and 24 hr (∼2% difference between all time points), suggests minimal incorporation into AA over the full time series, and that if host utilization of translocated ^13^C into FA lipogenesis occurs then it occurs around or after 120 hr. CEDP AA incorporation was lower than symbiotic anemones at 120 hr at 2.04% (Figure 4B). Incorporation into eicosapentaenoic/timnodonic acid, (C_20∶5_; EPA) at 120 hr was higher than AA in symbiotic anemones at 7.87% (± Std Dev 4.52) and in the CEDP at 13.36%. However, as with AA, although the increase was significant compared to earlier time points (*f*  = 5.25, P<0.05), there was no difference between 24 hr (3.12% ± Std Dev 0.76) to 72 hr (3.61% ± Std Dev 0.33). There was no additional ^13^C incorporation across any time points in docosatetraenoic/adrenic acid (C_22∶4_) synthesis in treated symbiotic anemones or CEDP (*f*  = 0.44, P>0.05; Figure 4D).

During synthesis of docosapentaenoic/clupanodonic acid (C_22∶5_, DPA) incorporation remained low (∼5%) with the exception of a significant spike above all other time points at 48 hr to 9.46% Std Dev 2.25 (*f*  = 6.12, P<0.05) (Figure 5A). The isotopic incorporation into the essential FA, docohexaenoic acid (C_22∶6_, DHA) appeared to be linear until a V_max_ at 72 hr (33.75% ± Std Dev 4.57) (Figure 5B). The CEDP DHA incorporation was greater than the 120 hr symbiotic anemones at 31.67%. Finally, two very long-chain FAs were identified within symbiotic anemones and CEDP samples (Figure 5C and D). Unusually, one of the very long-chain FA identified was an *n*–6 pathway derived product, octacosaheptaenoic acid (C_28∶7_; OHA) and not an *n*–3 concomitant pathway product, displaying a linear increase after 48 hr (significant after 72 hrs; *f*  = 178, P<0.001) reaching a maximum average incorporation of 24.95% at 120 hr and a CEDP incorporation of 27.94% (Figure 5C). The second very long-chain FA, octacosaoctaenoic acid (C_28∶8_; OOA), had the highest incorporation of all FAs with a V_max_ at 72 hr of 53.76% ± Std Dev 7.42 and is a *n*–3 pathway derived product (Figure 5D). The CEDP isotopic incorporation into the C_28∶8_ of 37.50% was higher than the C_28∶8_ incorporation within symbiotic anemones at 120 hr, which was significantly reduced from the incorporation at 72 hr (24.91% ± Std Dev 2.06; *f*  = 34.7, P<0.05). However, both of these uncommon FA displayed a similar but unique multinomial isotopologue distribution compared to the other FAs.

#### Transfer of methods between model systems

**Figure 6:**
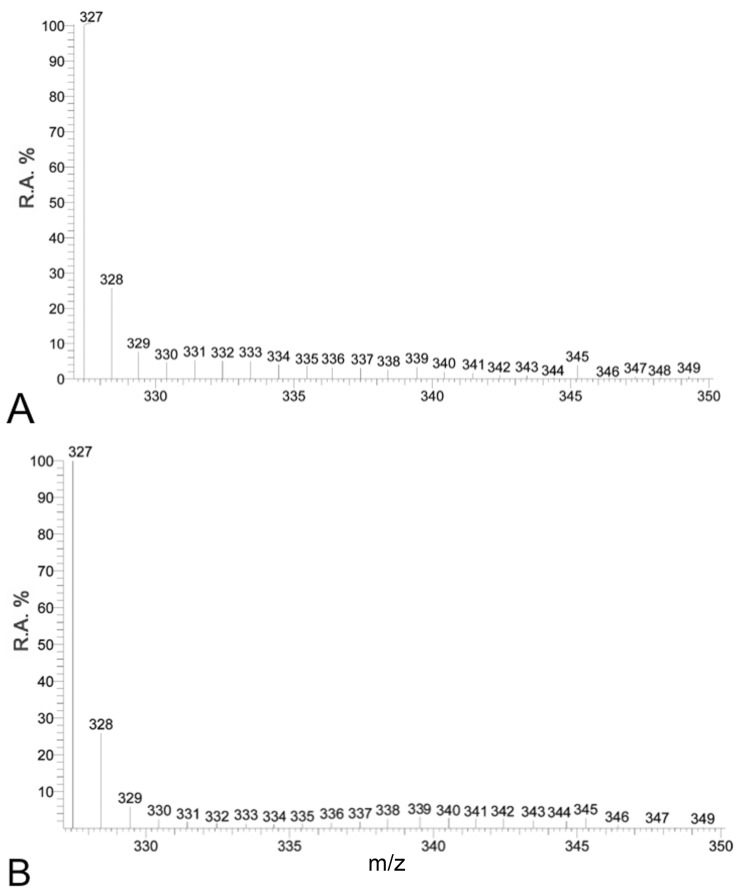
The mass spectrum profile of the fatty acid, DHA (C_22∶6, n–3_) within (A) *Acropora millepora* and (B) within a clade B dinoflagellate culture removed from the DI^13^C ASW treatments at 48 hr. (R.A.%  =  Relative Amount).

The transferability of the isotopic incorporation method to track the source of carbon to FA synthesis was shown to function in other model systems by the incorporation profiles of DHA (C_22∶6_) for the symbiotic coral, *Acropora millepora* (10.47% ± Std Dev 2.08; n = 3) (Figure 6A) and collated cultured clade B dinoflagellates (10.73%) (Figure 6B). The incorporation was below that of the symbiotic *A. pulchella* for the same time point (21.42% ± Std Dev 6.59) (Figure 5B).

## Discussion

**Figure 7:**
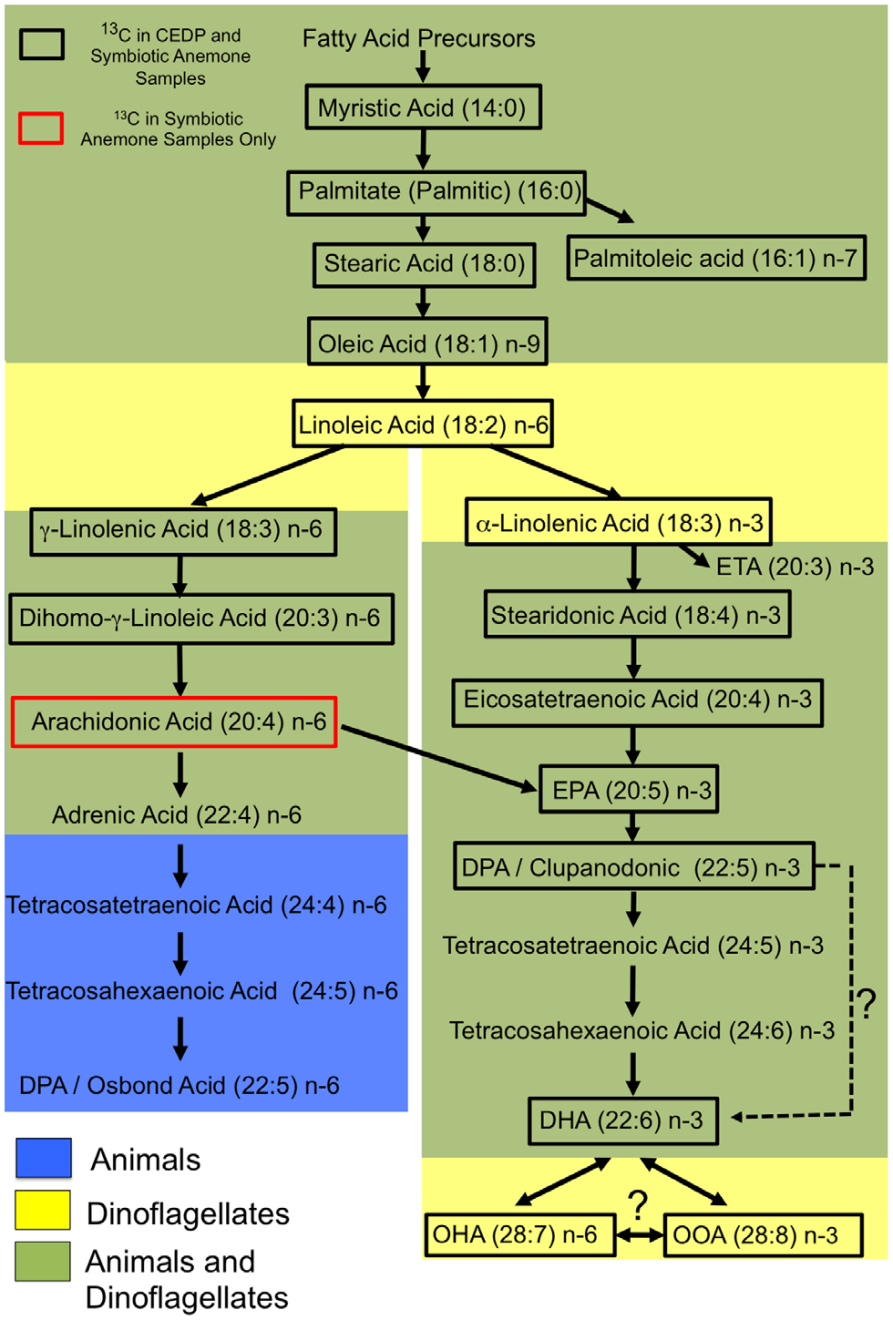
Schematic representation of the lipogenesis pathways utilizing DI^13^C (HCO_3_^−^) as a carbon source (dash arrow  =  potentially active and ?  =  links by evidence of incorporation, but not previously described) [**2**].

Fatty acids are synthesized from Acetyl CoA by the action of elongase enzymes that add 2 units of carbon at a time to form a carbon chain. A previously synthesised fatty acid of one type, for example, palmitic acid (C_16∶0_, 16 carbons and no double bonds), may be used as a foundation for the synthesis of the next product in a lipogenesis pathway, such as stearic acid (C_18∶0_, 18 carbons and no double bonds). Fatty acid (FA) chains may be reduced in carbon or altered further by a change in the number of double bonds by the action of other enzymes such as desaturases. For example, the monosaturated FA palmiteolic acid C _16∶1, *n*–7_ is synthesized from palmitic acid C_16∶0_ by the insertion of one double bond seven carbon atoms (*n*–7) from the terminal methyl group. This study applied a new method for tracking assimilation of a known carbon source into a variety of FAs. The method revealed intermediate products and their specific rates of synthesis within the cnidarian-dinoflagellate symbiosis that could be mapped into known FA lipogenesis pathways. The differential rates of known FAs synthesis generally aligned with the known lipogenesis pathways, and indicated candidate FAs, with potentially important functions within the symbiosis, in addition to transitory products for downstream metabolites. The potential lipogenesis of FAs and of unusual size and previously identified in other studies, such as the long-chain fatty acids (LCFA), OHA and OOA are discussed in more detail later. The findings of this study were unexpected in that the dinoflagellate symbiont, *Symbiodinium* sp. displayed a complex integration of both *n*–3 and *n*–6 LCFA pathways and not just *n*–3 “plant-like” pathways that were active throughout lipogenesis process (Figure 7). In addition, there was a surprising lack of evidence of host utilization of symbiont-derived FAs over a 120 hr incubation period, highlighted by the absence of additional ^13^C incorporation in late *n*–6 pathway lipogenesis. This was surprising in that it contravenes previous studies using ^14^C and an assumed automated translocation and utilization of photoassimilated metabolites in a mutualistic relationship regarding FA lipogenesis within the host [36], [37], [38], [39]. However, as mentioned in the introduction, although the influence of temporal variability on metabolite production and potential translocation should not be ignored [23] it equally needs to be put into perspective that different metabolic and cell division rates occur in both host and symbiont cells, with respect to the metabolites being produced, where they are utilized and if they are actually available for potential translocation.

The main model system used was the anemone, *Aiptasia pulchella* together with its clade B1 *Symbiodinium* sp. dinoflagellate symbiont. The method’s transferability was demonstrated using other model systems, however as cross-model optimization was not the focus of this study, we suggest targeted method adaptation associated with specific model use, to enhance specific isotopic incorporation depending upon the required application. The efficiency of FA extraction method was not significantly different between aposymbiotic and symbiotic anemones samples. This method, unlike previous symbiotic cnidarian lipid studies [9], [13], [14], [40] enables a measure of extraction efficiency to be calculated and, as such, could be applied in future studies for inter-study comparison. The recovered FAs from sample total lipids were consistent between symbiotic and aposymbiotic samples, but potentially amounted to less than half of the available FAs. In future, the efficiency of the extraction method could be potentially improved by the use of additional steps, such as sonication or the heating of solvents. The naturally occurring abundance of isotopologues with 1–2% ^13^C [13], [30] represented by 1 or 2 Da above the monoisotopic FA ion (pure ^12^C) in the MS spectrum were present in all samples (Figure 1, 2, 3, 4, 5, and 6). Differential amounts and rates of increased ^13^C incorporation in FAs from enrichment could indicate that either, priority is given to carbon allocation for certain FAs over others, and/or the rates of production are affected by synthesized or catabolized downstream products. The influence of these processes, alternative sources of carbon, potential turnover rates or storage capacity should be taken into account when examining individual FA profiles and potential functions as was done here, and noted in other isotope ratio studies [41].

Throughout the incubation period of enriched ^13^C stable isotope used in this study, the incorporation of ^13^C during FA lipogenesis was only detected in symbiotic anemones and within the combined expelled dinoflagellate pellets (CEDP’s) from different anemones. ^13^C incorporation varied in both sample types with different FAs, and the different profiles of isotopic incorporation over time proceeding down the cascading lipogenesis pathways is likely to be a result of both transfer from shorter to longer chain synthesis and catabolism of each metabolite. For example, the isotopic incorporation profile of myristic acid (C_14∶0_) was characterised by a rapid linear increase followed by a plateau (Figure 2A). The plateau may either indicate storage or utilization in secondary metabolite synthesis. The latter and potentially more significant within the symbiosis is the potential use of myristate in covalent attachment to the N-terminal of proteins known as *N*-myristoylation. The *N*-myristoylated proteolipids have important roles in membrane and cellular signal transduction cascades, and protein-protein interactions, including apoptosis and pathogenesis response, such as endothelial nitric-oxide synthase (NOS) and GTPase regulation [1]. These processes have previously been associated with regulation of cnidarian-dinoflagellate symbiosis [42].

The linear incorporation profile of palmitic acid (C_16∶0_) (Figure 2B) was reciprocated in the conversion of a proportion into palmiteolic acid (C_16∶1, *n*–7_) (Figure 2C). C_16∶0_ is one of the most abundant FAs, a precursor to lipogenesis and in combination with C_16∶1_ represent two of the main components of total lipids in the cnidarian-dinoflagellate consortium [9], [14], [40], [43]. The consistent linear incorporation profile into C_16∶0_ correlated with the continuous synthesis and turnover of a large quantity of FA for other products, as shown with a consistent increase in ^13^C as ^12^C supplies and subsequent incorporation were reduced over time. The C_16∶1_ profile aligns with the C_16∶0_ explanation for synthesis as this is a direct downstream derivative of C_16∶0_. However, the function within the symbiosis remains unknown.

In tracking lipogenesis pathways the saturated FA synthesized from C_16∶0_ is stearic acid (C_18∶0_), displayed a profile indicative of an intermediate undergoing rapid turnover into downstream oleic acid (C_18∶1_) (Figure 2D and 3A). The subsequent high incorporation within linoleic acid (C_18∶2_) and linolenic acid (C_18∶3_) from symbiotic anemones and CEDP followed the *n*–3 lipogenesis pathway, which is found within marine algae (Figure 3B and C). The C_18∶2, *n*–6_ to C_18∶3_ conversion is a potential diversion point for pathways of FA lipogenesis. The incorporation into either α-linolenic C_18∶3, *n*–3_ or γ-linolenic C_18∶3, *n*–6_ from C_18∶2, *n*–6_, was not examined, although both isomers were detected. However, by tracking downstream incorporation to either arachidonic acid (C_20∶4, *n*–6_) or stearidonic acid (C_18∶4, *n*–3_), the active pathway(s) for further incorporation can be identified. In keeping with this assumption, incorporation was detected within C_18∶4, I–3_ (Figure 3D) and dihomo-γ-linoleic acid (C_20∶3, *n*–6_), indicating both further *n*–3 and *n*–6 activity in both the symbiotic anemones and CEDP (Figure 4A). The evidence of a division of DI^13^C resource between *n*–3 and *n*–6 pathways during long-chain FA synthesis, was only apparent within the dinoflagellate, which was reciprocated within the CEDP samples up until and including incorporation into C_20∶4, *n*–6_ at around 120 hr. Multiple pathway long-chain fatty acid lipogenesis have previously been proposed and shown in other algae [44], [45], [46], and appears to be also active within the dinoflagellate *Symbiodinium* sp. A proposed pathway interaction of these pathways as a result of this study is shown in Figure 7. Although *n*–6 FAs were detected continuously along the pathway up and including until adrenic acid (C_22∶4_) within the dinoflagellates, the incorporation was only detected up to arachidonic acid (C_20∶4_) which may link back across to the *n*–3 pathway and EPA (C_20∶5 *n*–3_) and onwards downstream (Figure 7). There was no indication of direct utilization of DI^13^C in further *n*–6 downstream products, including and beyond docosatetraenoic/adrenic acid (C_22∶4, n–6_), which would normally be attributed to host/animal only (Figure 4D).

The remaining downstream FAs with incorporation were associated with the *n*–3 lipogenesis pathway (within the dinoflagellate) including eicosapentaenoic acid (EPA, C_20∶5_) and docosapentaenoic/clupanodonic acid (DPA; C_22∶5_), which appeared to have little incorporation. Such delayed incorporation may indicate either a rapid turnover and thus low accumulation, or that synthesis is retarded by a potential negative feedback loop via docosahexaenoic acid (DHA; C_22∶6_) concentration, which is synthesized via the intermediate DPA; C_22∶5_ (Figure 5A). The potential activity of a negative feedback loop control on EPA production correlates with the decrease in DHA at 120 hr. However, no control loop has previously been described and requires further research. The high incorporation into the symbiotic anemone’s C_22∶6_ up until 72 hr followed by a significant decrease at 120 hr is possibly due to feedback control or resource limitation. The CEDP C_22∶6_ incorporation remained within the range of the symbiotic anemones, again suggesting the dinoflagellates were responsible for DI^13^C utilization (Figure 5B). The evidence presented here of high incorporation and accumulation in end *n*–3 pathway essential FAs, such as DHA indicated a priority to produce large amounts of these products and correlates with similar production and accumulation in previous studies [8], [9], [14], [43].

The importance of *n*–3 derived essential FAs such as DHA, to supplement animal diets is well known [3]. A large supply of essential FAs may also be important in symbiosis regulation and could correlate with a proportion of DHA conversion to very LCFA, such as octacosaoctaenoic acid (C_28∶8, *n*–3_). The multinomial peak profile of isotopologues indicated ^13^C incorporation was skewed towards both high and low content isotopologues. The latter indicated use of ^13^C in elongation of a pool of pre-treatment non-enriched, normally proportioned ^12^C/^13^C DHA isotopologues (*m/z* 327) to OOA (C_28∶8, n–3_; *m/z* 407) to form a series of isotopologues from *m/z* 328 to *m/z* 413 (6×^13^C added during elongation of DHA) (Figure 5D). The presence of OOA in dinoflagellates is already known, although the synthesis and novel adaptation to the *n*–3 pathway was unknown [47]. The ^13^C incorporation of OOA was the highest of all fatty acids in the symbiotic anemones and although the function remains unknown, OOA could lead to downstream metabolites or storage whereby later catabolism leads to additional DHA. The description of the interaction between DHA and OOA (C_28∶8, *n*–3_) is further complicated by the incorporation into octacosaheptaenoic acid (OHA; C_28∶7, *n*–6_) (Figure 5C) which displays the similar multinomial peak of the C_28–8,*n*–3_. The less pronounced peak distribution of this *n*–6 LCFA, indicated that it is either an intermediate between the *n*–3 DHA and the LCFA, OOA (C_28∶8, *n*–3_), or a direct derivative of one of the two and therefore indicating a direct and close interaction between *n*–3 and *n*–6 enzyme activity, which is independent of upstream pathway integration (Figure 7). The functions of these particular highly produced essential FAs, OOA and OHA within this symbiosis and dinoflagellates are unknown and are currently the focus of further research.

The absence of incorporation in enriched DI^13^C treated aposymbiotic control anemones and non-^13^C ASW treated symbiotic anemones (Figure 1A-B), combined with finding that the ^13^C-fatty acid profiles of CEDPs and symbiotic anemones do not differ greatly, suggests symbiotic dinoflagellates are essential for DI^13^C incorporation into the FAs of symbiotic anemones. In support of this conclusion is the finding that all FAs found to incorporate ^13^C can be assembled as a schematic pathway that aligns with *n*–3 and *n*–6 algal lipogenesis (Figure 7). Although late *n*–6 pathway FA were detected within both aposymbotic and symbiotic anemone hosts, there was no direct evidence of DI^13^C derived FA synthesis or processing of endosymbiont derived FAs within the cnidarian host. An alternative explanation for the findings is that symbiont derived FAs were translocated to the host, but not used in host FA synthesis and rapidly catabolized into undetectable non-FA metabolites, such as ATP. Whilst this is a possibility and cannot be dismissed, it is also unlikely as there was no evidence of the host free FAs, including downstream longer chain FAs, containing enriched isotopic incorporation. If symbiont derived FA or derived catabolized carbon were made available to the host it is unlikely that they would be discriminated against for incorporation for host FA elongation and lipogenesis due to the important nature of these metabolites.

The difference in rates of carbon fixation as a result of the symbiosis that have been reported in ^14^C FA incorporation studies and this ^13^C study are quite considerable, and raises questions as to where the radioisotope FA signals that were detected were actually located and if there was considerable symbiont contamination involved? In respect to this, attempts were made in independent trials (data not shown) to obtain algal contaminate-free samples using this highly sensitive technique and it was concluded that obtaining symbiotic host fractions that were free of symbiont FA contamination (as indicated by the presence of LCFAs) was not consistently possible. The consensus of evidence from this study suggests that DI^13^C derived FAs via the symbiotic dinoflagellate were not used directly or utilized via potential symbiont derived triacylglycerol stores in host FA lipogenesis during the treatment period. Further, if metabolites, including lipids and FAs produced through the symbiosis help meet 90–99% of host energy requirements [15], [19] and the respective lipid energy stores are derived from dinoflagellate photosynthates [8] then the results of this study imply either that the utilization of DIC in host FA lipogenesis is subjected to a 120 hr (5 day+) lag period or that the required carbon is derived from an alternative source. DI^13^C incorporation profiles were mainly below 50% and clearly showed ^12^C still being incorporated by the dinoflagellate, suggesting additional sources of carbon are important such as host respiration and/or heterotrophy are important [12], [28]. Host heterotrophy influences partner lipid content and can provide 35% of the daily metabolic requirements of healthy symbiotic corals, and 100% in bleached individuals [12], [29]. The fact that anemones were not fed during the experimental treatment could explain why some FA synthesis declined between 72 hr and 120 hr, and is potentially in response to reduced supplies of a limiting resource, such as nitrogen for the symbiont in the form of ammonia via host catabolism [19]. The transferable enriched isotopic incorporation method presented here clearly demonstrated how one form of carbon is differentially utilized and distributed during FA lipogenesis in cnidarian-dinoflagellate symbiosis. This has important connotations for understanding both the health and integrity of the individual dinoflagellate and the symbiotic relationship with the cnidarian host.

## Supporting Information

Figure S1 **(A). The total quantity of AA (arachidonic acid; C_20∶4, n–6_) within symbiotic and aposymbiotic** ***A. pulchella*** **used to calculate the extraction efficiency from a mixture of both non-enriched and enriched DI^13^C media at 72 hr was not significantly different between symbiotic (1.029 µg/mg of tissue ± Std Dev 0.138) and aposymbiotic anemones (0.944 µg/mg of tissue ± Std Dev 0.279) (** ***t*** **_(6)_  = 0.56, P  = 0.6).** (B) The fatty acid extraction efficiency from total lipid extractions from symbiotic and aposymbiotic *Aiptasia pulchella*. There was no significant difference in extraction efficiency (*t*_(6)_ = 0.96, P  = 0.37) between aposymbiotic (43.01% ± Std Dev 12.19) and symbiotic *A. pulchella* (48.97% ± Std Dev 10.46) (n = 6, Error bars =  Std Dev).
(TIF)

Table S1 **The HPLC gradient (%) for analysis of lipid extracts.**
(DOC)

Table S2 **Retention times and** ***m/z*** **of Fatty Acid Standards as determined in this study (Sapphire Bioscience, Waterloo, NSW, Australia).**
(DOCX)

Table S3 **The adjusted HPLC gradient (%) for quantitation analysis of lipid extracts.**
(DOCX)
