# Supplementary figures and images for: A Lipidomic Approach to Understanding Free Fatty Acid Lipogenesis Derived from Dissolved Inorganic Carbon within Cnidarian-Dinoflagellate Symbiosis
Source: PLoS One. 2012 Oct 24;7(10):e46801. doi: 10.1371/journal.pone.0046801 (PMC3480374; doi:10.1371/journal.pone.0046801)

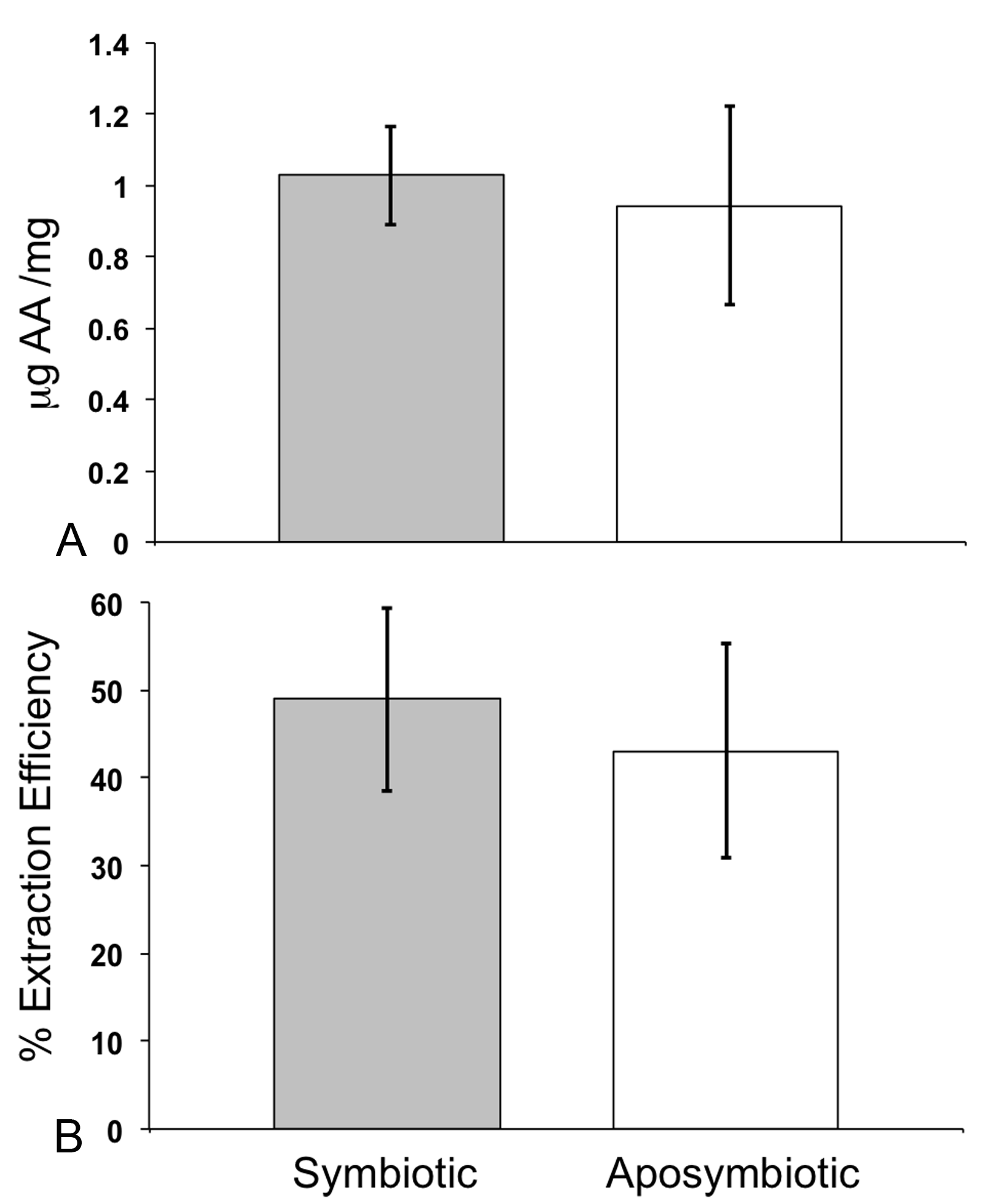

Supplement: Figure S1 — (A). The total quantity of AA (arachidonic acid; C20∶4, n–6) within symbiotic and aposymbiotic A. pulchella used to calculate the extraction efficiency from a mixture of both non-enriched and enriched DI13C media at 72 hr was not significantly different between symbiotic (1.029 µg/mg of tissue ± Std Dev 0.138) and aposymbiotic anemones (0.944 µg/mg of tissue ± Std Dev 0.279) ( t (6) = 0.56, P = 0.6). (B) The fatty acid extraction efficiency from total lipid extractions from symbiotic and aposymbiotic Aiptasia pulchella. There was no significant difference in extraction efficiency (t (6) = 0.96, P = 0.37) between aposymbiotic (43.01% ± Std Dev 12.19) and symbiotic A. pulchella (48.97% ± Std Dev 10.46) (n = 6, Error bars = Std Dev). (TIF) [file pone.0046801.s001.tif]
