# Supplementary material for: A Lipidomic Approach to Understanding Free Fatty Acid Lipogenesis Derived from Dissolved Inorganic Carbon within Cnidarian-Dinoflagellate Symbiosis
Source: PLoS One. 2012 Oct 24;7(10):e46801. doi: 10.1371/journal.pone.0046801 (PMC3480374; doi:10.1371/journal.pone.0046801)
Supplement: Table S1 — The HPLC gradient (%) for analysis of lipid extracts. (DOC) [file pone.0046801.s002.doc]

| **Time (Min)** | **0** | **15** | **17.5** | **18** | **26** | **27** | **34** | **40** |  |
| --- | --- | --- | --- | --- | --- | --- | --- | --- | --- |
| A (MilliQ H_2_0) | 40 | 20 | 15 | 5 | 5 | 0 | 40 | 40 |  |
| B (Acetonitrile) | 60 | 80 | 85 | 95 | 95 | 100 | 60 | 60 |  |

Supplementary Table 1: The HPLC gradient (%) for analysis of lipid extracts.
