# Supplementary material for: A Lipidomic Approach to Understanding Free Fatty Acid Lipogenesis Derived from Dissolved Inorganic Carbon within Cnidarian-Dinoflagellate Symbiosis
Source: PLoS One. 2012 Oct 24;7(10):e46801. doi: 10.1371/journal.pone.0046801 (PMC3480374; doi:10.1371/journal.pone.0046801)
Supplement: Table S2 — Retention times and m/z of Fatty Acid Standards as determined in this study (Sapphire Bioscience, Waterloo, NSW, Australia). (DOCX) [file pone.0046801.s003.docx]

| **Fatty Acid Common Name and (*Systematic name*)** | **Fatty Acid Signature**  **Standards** | **Mass / Charge**  **(*m/z*)** | **Retention Time (±0.5 min)** |
| --- | --- | --- | --- |
| Myristic Acid (*Tetradecanoic acid*) | 14 : 0 | 227 | 13.39 |
| Palmitic Acid (*Hexadecanoic acid*) | 16 : 0 | 255 | 20.06 |
| Palmitoleic Acid (*Hexadecenoic acid*) | 16 :1 | 253 | 15.09 |
| Stearic Acid (*Octadecanoic acid*) | 18: 0 | 283 | 23.81 |
| Oleic Acid (*Octadecenoic acid*) | 18: 1 (n-9, cis) | 281 | 20.72 |
| Elaidic Acid (*Octadecenoic acid*) | 18: 1 (n-9, trans) | 281 | 21.15 |
| Linoleic Acid | 18: 2 (n-6) | 279 | 16.9 |
| Linoleic Acid (*Conjugated*) | 18: 2 10E, 12E (trans) | 279 | 17.33 |
| Linoleic Acid (*Conjugated*) | 18: 2 9Z, 11E | 279 | 17.54 |
| Linoleic Acid (*Conjugated*) | 18: 2 (n-9) 9E 11E | 279 | 18.45 |
| α-Linolenic Acid | 18: 3 (n-3) | 277 | 12.93 |
| γ-Linolenic Acid | 18: 3 (n-6) | 277 | 13.55 |
| Stearidonic / Parinaric Acid (*Octadecatetraenoic acid*) | 18: 4 | 275 | 9.76 |
| Arachidic Acid (*Eicosanoic acid*) | 20 :0 | 311 | 29.28 |
| Gadoleic acid (*Eicosenoic acid*) | 20: 1 | 309 | 24.15 |
| *Eicosadienoic acid* | 20: 2 | 307 | 21.28 |
| *Eicosatrienoic acid* | 20: 3 (n-3) | 305 | 18.83 |
| *Eicosatrienoic acid* | 20: 3 (n-6) | 305 | 18.94 |
| *Eicosatrienoic acid* | 20: 3 (n-9) | 305 | 20.03 |
| Arachidonic Acid (*Eicosatetraenoic acid*) | 20: 4 (n-3) | 303 | 15.12 |
| Arachidonic Acid (*Eicosatetraenoic acid*) | 20: 4 (n-6) | 303 | 16.06 |
| Timnodonic Acid (*5, 8, 11, 14, 17-Eicosapentaenoic Acid : EPA*) | 20: 5 | 301 | 12.24 |
| Behenic Acid (*Docosanoic acid*) | 22: 0 | 339 | 26.00 |
| Erucic Acid (*Docosenoic acid*) | 22: 1 | 337 | 29.38 |
| *Docosatrienoic acid* | 22: 3 | 333 | 22.25 |
| Adrenic Acid (*Docosatetraenoic acid*) | 22: 4 | 331 | 20.34 |
| Clupanodonic Acid (*7, 10, 13, 15, 19- Docosapentaenoic Acid : DPA*) | 22: 5 | 329 | 17.22 |
| *4,7,10, 13, 16, 19-Docosahexaenoic Acid : DHA* | 22: 6 | 327 | 14.63 |

Supplementary Table 2:
