# Supplementary material for: A Lipidomic Approach to Understanding Free Fatty Acid Lipogenesis Derived from Dissolved Inorganic Carbon within Cnidarian-Dinoflagellate Symbiosis
Source: PLoS One. 2012 Oct 24;7(10):e46801. doi: 10.1371/journal.pone.0046801 (PMC3480374; doi:10.1371/journal.pone.0046801)
Supplement: Table S3 — The adjusted HPLC gradient (%) for quantitation analysis of lipid extracts. (DOCX) [file pone.0046801.s004.docx]

| **Time (Min)** | **0** | **5** | **9** | **9.2** | **15** |
| --- | --- | --- | --- | --- | --- |
| A (MilleQ H_2_0) | 20 | 0 | 0 | 20 | 20 |
| B (Acetonitrile) | 80 | 100 | 100 | 80 | 100 |

Supplementary Table 3:
